# Mycorrhizal inoculation and fertilizer microdosing interactions in pearl millet (*Pennisetum glaucum*) under greenhouse conditions

**DOI:** 10.3389/ffunb.2024.1448156

**Published:** 2024-08-29

**Authors:** Malick Ndiaye, Alain Mollier, Adama Diouf, Tahir Abdoulaye Diop

**Affiliations:** ^1^ Laboratoire de Biotechnologies des Champignons, Département de Biologie Végétale, Université Cheikh Anta Diop, Dakar, Senegal; ^2^ UMR 1391 Interactions Sol Plant Atmosphère (ISPA), Institut National de Recherches pour l’Agriculture, l’Alimentation et l’Environnement (INRAE), Bordeaux Sciences Agro, Bordeaux, France; ^3^ Laboratoire Commun de Microbiologie, Institut de Recherche pour Développement (IRD)/Institut Sénégalais de Recherches Agricoles (ISRA)/ Université Cheikh Anta Diop de Dakar (UCAD), Centre de recherche de Bel Air, Dakar, Senegal; ^4^ Polytech Diamniadio, Département Sciences et Techniques Agricoles, Alimentaires et Nutritionnelles, Université Amadou Mahtar Mbow, Dakar, Senegal

**Keywords:** arbuscular mycorrhizal fungi, pearl millet, mineral nutrient, microdosing fertilizer, manure

## Abstract

**Introduction:**

Soil fertility is a major constraint to agricultural development in the Sahel region of Africa. One alternative to reducing the use of mineral fertilizers is to partially replace them with microbes that promote nutrition and growth, such as arbuscular mycorrhizal fungi (AMF). Mineral fertilizer microdosing is a technique developed to enhance fertilizer efficiency and encourage smallholder farmers to adopt higher mineral fertilizer applications.

**Methods:**

A pot experiment was set up to study the effects of AMF inoculation on the mineral nutrition of pearl millet under mineral fertilizer microdosing conditions. The experimental setup followed a randomized complete block design with five replicates. The treatments tested on millet were an absolute control and eight microdoses derived from the combination of three doses of 15- 10-10 [nitrogen, phosphorus, and potassium (NPK)] mineral fertilizer (2 g, 3 g, and 5 g per pot), three doses of urea (1 g, 2 g, and 3 g per pot), and three doses of organic manure (OM) (200 g, 400 g, and 600 g), combined with and without AMF (Rhizophagus irregularis and Rhizophagus aggregatum). The parameters studied were growth, root colonization by AMF, and mineral nutrition. Plant height, stem diameter, root dry biomass, and percentage of root mycorrhization were measured.

**Results and discussion:**

The results revealed a significant effect of the fertilizers on the growth of pearl millet compared to the control. AMF and OM treatments resulted in the highest biomass production. AMF combined with microdoses of NPK improved N and calcium (Ca) concentrations, while their combination with organic matter mainly improved the K concentration. Combining AMF with microdosed NPK and compost enhanced zinc (Zn) and nickel (Ni) concentrations. Root colonization varied from 0.55 to 56.4%. This investigation highlights the positive effects of AMF inoculation on nutrient uptake efficiency when combined with microdosing fertilization.

## 1 Introduction

Pearl millet plays an important role in the lives of smallholder farmers in West Africa, serving as a staple food, grain, animal feed, fuel, and construction material (Pucher et al., 2014; Passot et al., 2016; Ndour et al., 2021). It is highly adapted to hot climates with low annual rainfall (300–500 mm) and poor sandy soils, thriving where other crops fail (Supriya et al., 2011; Vadez et al., 2012; Prasad et al., 2017; Ullah, 2017; Faiz et al., 2022). Its production is important for providing significant calorie intake (Serba and Yadav, 2016; Djanaguiraman et al., 2018; Debieu et al., 2018; Diatta et al., 2020).

Despite its significance, pearl millet yields in farming environments remain low, averaging less than 1000 kg ha^−1^ (Faye et al., 2023). The main constraints to increasing millet production in Senegal include climate change factors such as erratic rainfall patterns, high temperatures, and low soil fertility (Haussmann et al., 2007; Affholder et al., 2013; Defrance et al., 2020; Monerie et al., 2020; Stewart et al., 2020). Additionally, the reliance on rain-fed agriculture, and economic constraints faced by smallholder farmers who have limited incentives to purchase mineral fertilizers, exacerbate production uncertainty and food insecurity (Ganyo et al., 2019; Tounkara et al., 2020; Bastos et al., 2022). Further, the inappropriate use of mineral fertilizers has also led to reduced yields and declining soil fertility (Simpson et al., 2011; Ibrahim et al., 2015). Excess chemical fertilizers can accumulate in the soil (Selim, 2020; Pan et al., 2021), causing soil toxicity, deteriorating soil quality, depleting beneficial soil microflora, and leading to the leaching of toxic elements into groundwater and runoff into water resources (Chaudhary et al., 2017; Meena et al., 2020; Schaefer et al., 2021; Shanmugavel et al., 2023). To improve the efficient use of the mineral fertilizer, the microdosing technology developed by the International Crops Research Institute for the Semi-Arid Tropics (ICRISAT) has shown promising results in enhancing millet yields in the Sahel region of Africa (Aune and Bationo, 2008; Ibrahim et al., 2015; Blessing et al., 2017; Saba et al., 2017; Manssour et al., 2020). This technology involves applying a small amount of mineral fertilizer to the crop seeds at sowing or a few weeks after planting (Aune and Bationo, 2008; Twomlow et al., 2010; Saba et al., 2017). Nevertheless, the low soil organic matter in Sahelian sandy soils limits the effectiveness of this approach (Ibrahim et al., 2015). Therefore, to enhance crop response to fertilizer microdosing, it is essential to combine this method with the application of organic matter (Twomlow et al., 2010).

However, in the context of sustainable agriculture aimed at reducing the use of chemical fertilizers, agricultural productivity increasingly relies on the interactions between soil and microbiota (Thirkell et al., 2017; Genre et al., 2020; Ndour et al., 2021; Adoko et al., 2022). The use of microbial biostimulants in agriculture represents a natural and environmental friendly alternative for enhancing productivity (Fasusi et al., 2021). Several studies have demonstrated the huge potential of plant-associated microorganisms to improve plant nutrition and yield particularly under greenhouse conditions (Pellegrino et al., 2015; Schütz et al., 2018; Syed Ab Rahman et al., 2018; Zhang et al., 2019; Compant et al., 2019). One effective strategy for boosting plant growth and mineral nutrition is to inoculate plants with arbuscular mycorrhizal fungi (AMF). These fungi are integral components of soil and plant roots forming a symbiosis with many cereal crops (Smith and Smith, 2011). AMF enhance plant nutrient acquisition, especially phosphorus (P), in low P soils, by utilizing a symbiotic Pi uptake pathway (Bucher, 2007). This symbiosis improves P uptake efficiency, reducing the need for chemical fertilizers and mitigating potential environmental losses (Adesemoye et al., 2008; Alori and Babalola, 2018; Ghobadi et al., 2020; Trejo et al., 2021; Schaefer et al., 2021). Therefore, understanding the interactions between microbes, fertilizers, and plants is crucial for optimizing these practices (Adesemoye and Kloepper, 2009; Wang et al., 2017; Suman et al., 2022). Several studies have highlighted the benefits of double inoculation with AMF and nitrogen, phosphorus, and potassium (NPK) for plant growth (Adesemoye and Kloepper, 2009; Tshibangu Kazadi et al., 2020; Ziane et al., 2021; Fall et al., 2023; Mulyadi and Jiang, 2023). However, the effects of double inoculation with AMF and fertilizer microdosing on pearl millet in Senegal have not been explored. This study aims to examine the interactions between double AMF inoculation and NPK fertilizer application on nutrient absorption and biomass production in pearl millet. We hypothesize that combining AMF inoculation with NPK microdosing will enhance the plant’s mineral nutrient status and biomass. This study, therefore, sets out to identify optimal microdosing rates for pearl millet in combination with AMF.

## 2 Materials and methods

### 2.1 Soil

The experiment was carried out at the Department of Plant Biology, Cheikh Anta Diop University, Senegal. The soil used in this study was collected at the end of the rainy season from Touba Toul at 5–20 cm depth (70 km from Dakar, Senegal). Soil characteristics are detailed in **Table 1**. The average nutrient content of cow manure, measured at 12% moisture, was 2.28% potassium, 18.64% total carbon, 0.96% total nitrogen, and 0.29% total phosphorus.

**Table 1:** Physico-chemical properties of soil.

Component	Content
Clay	3.6
Silt	1.6
Fine silt	2.9
Fine sand	51
Coarse sand	40.9
Conductivity 1/10 µs/cm	65
% C	1.57
% MO	1.24
% N	0.11
C/N	11
Total phosphorus (ppm)	47
Available phosphorus (ppm)	3.1
pH (sol/KCl ratio 1:2) 4.5	4.6
pH (sol/KCl ratio 1:2) 4.5	4.6

### 2.2 Mycorrhizal inoculum

Mycorrhizal inocula containing indigenous species (*Rhizophagus irregularis* and *Rhizophagus aggregatum*) were obtained from the Laboratory of Fungal Biotechnology (LBC) of the Department of Plant Biology, Cheikh Anta Diop University, Senegal, and multiplied using maize as the host plant. The AMF inoculants used in the experiment were prepared by inoculating sandy soils with spores of *Rhizophagus irregularis* and *Rhizophagus aggregatum* separately along with extraradical mycelium generated in pot cultures by maize plants. Spores had an average density of 40 spores per gram of soil per pot. The final inoculum applied was a mixture of both species in equal proportions, resulting in an average density of 80 spores per gram and 85% root infection.

### 2.3 Experimental design and growth conditions

Traditional pearl millet variety Souna 3 was selected due to its widespread adaptation and preference among smallholder farmers in Senegal (Bastos et al., 2022). The seeds were sown in pots, each containing 3 kg of sandy soil (**Table 1**), in January. The millet plants were inoculated, and organic matter was added at the time of sowing. One week after germination, the plants were thinned to two per hill. A factorial complete randomized design was used, with treatments including fertilizer with or without AMF inoculum, and five replicates (**Table 2**). The average nutrient content of cow manure, at 12% moisture, was 2.28% potassium, 18.64% total carbon, 0.96% total nitrogen, and 0.29% total phosphorus. NPK (15-10-10) was applied 10 days after emergence, followed by urea (U, 46% N) 1 month later. The microdosing rates were 0, 0.2, 0.4, 0.6, and 0.8 g per pot, corresponding to 0, 5, 10, 15, and 20 kg ha^−1^. Plants were inoculated with AMF by adding 20 g of inoculum directly to the roots, while the control received 20 g of sterile soil. The seedlings were grown from January to March in a greenhouse with an average day/night cycle of 12/12 hours, temperatures of 32/25°C, and 40-50% air humidity. Plants were watered with tap water and harvested after 3 months.

**Table 2:** List of treatments.

Treatment	Description
T0	Sandy soil (control)
(NPK)_3_U_2_	3g NPK and 2g urea/poquet
OM_600_	600 g/poquet OM
AMF	AMF
(NPK)_3_U_2_+AMF	3g NPK and 2g urea/poquet + AMF
OM_600_+AMF	600 g/poquet OM + AMF
(NPK)_2_U_1_+OM_200_+AMF	2g NPK and 1g urea + 200 g OM/poquet + AMF
(NPK)_3_U_2_+OM_400_+AMF	3g NPK and 2g urea/poquet +400 g OM/poquet + AMF
(NPK) _5U4_+OM_600_+AMF	5g NPK and 4g urea + 600g OM/poquet + AMF

The indices for the treatment names indicate the amount of NPK fertilizer, urea, and cow manure (OM) added per pot (g).

### 2.4 Measurements and chemical analysis

The height and stem diameter of the plants were measured using a graduated ruler and caliper. Twelve weeks after sowing, shoots and roots were harvested separately. Fresh root subsamples were taken to assess root colonization rates. Roots were initially rinsed with tap water and then thoroughly washed with distilled water. The percentage of mycorrhizal root infection was determined by microscopic observation of fungal colonization after clearing the washed roots in 10% KOH and staining with 0.05% trypan blue in lactophenol (v/v), following the method described by Phillips and Hayman (1970). Relative arbuscular richness (i.e., the percentage of arbuscules among AMF structures in the roots) was calculated using the grid-line intersect method (Giovannetti and Mosse, 1980). Root and shoot dry weights were measured after oven-drying at 55°C for 96 hours. Plant analyses were conducted at the UMR Interaction Soil Plant Atmosphere (ISPA) of the National Research Institute for Agriculture, Food and the Environment (INRAE) in France. The dried shoots were ground, and foliar nutrient contents were assessed after digestion with nitric acid (HNO_3_) + hydrogen peroxide (H_2_O_2_). Elements were determined using Inductively Coupled Plasma (ICP). A 500 mg sample of dried powder was mixed with 5 mL of an HNO_3_/H_2_O_2_ mix (4:1, v/v) in a 50 mL Teflon ^®^ tube and placed in a graphite digestion block (DigiPREP MS, SCP Science). The samples were then filtered, and the total concentrations of P, K, calcium (Ca), magnesium (Mg), zinc (Zn), copper (Cu), manganese (Mn), itron (Fe), and nickel (Ni) in the plant digests were measured by inductively coupled plasma atomic emission spectroscopy (ICP-OES) (ICAP 6300, Thermo Fischer). The nitrogen concentration in leaf samples was analyzed using Dynamic Flash Combustion technology (Thermo Flash, EA 1112).

### 2.5 Statistical analysis

Statistical procedures were conducted using R software version 3.4.4. Data were first tested for normality and homogeneity using the Shapiro-Wilk test. One-way or two-way analysis of variance (ANOVA) was performed to partition the variance into the main effects and interactions between inoculation and fertilization. Significantly different means were identified using the Tukey’s Honestly Significant Difference (HSD) test at a 5% probability level. Results are presented with p < 0.05 considered significant.

## 3 Results

### 3.1 Effects of treatments on growth parameters

The treatments significantly affected growth parameters (**Table 3**). The control treatment exhibited the lowest growth parameters, while the highest growth was observed with the AMF treatment. The tallest plants were recorded with the AMF treatment (107 cm) and OM_600_ treatment (102 cm), whereas the control had the shortest plants. No significant differences were found among the following combined treatments: (NPK)_3_U_2_+AMF; OM_600_+AMF; OM_200_+AMF; (NPK)_3_U_2_+OM_400_+AMF and (NPK)_5_U_4_+OM_600_+AMF. Variations in shoot dry weight followed the same trends as plant height.

**Table 3:** Effect of AMF–NPK fertilizer combinations on plant height (cm), stem diameter (mm), panicle length (cm), dry weight (g), and root AMF colonization in pearl millet.

Treatment	Plant height (cm)	Stem diameter (mm)	Panicle length (cm)	Shoot dry weight (g)	AMF colonization (%)
T0	32.3 ± 9.1 ^d^	2.7 ± 0.8 ^e^	5.1 ± 0.7 ^c^	0.2 ± 0.1 ^d^	0.0 ^d^
(NPK)_3_U_2_	67.7 ± 5.0 ^c^	5.50 ± 1.29 ^d^	17.8 ± 6.5 ^ab^	3.2 ± 1.67 ^bcd^	0.5 ^c^
OM_600_	102.3 ± 15.4 ^ab^	9.8 ± 1.6 ^a^	19.2 ± 5 ^ab^	8.1 ± 5.1 ^abc^	0.0 ^d^
AMF	106.8 ± 14.2 ^a^	8.8 ± 1.8 ^ab^	25.2 ± 10.1 ^a^	11.7 ± 5.8 ^a^	56.4 ^a^
(NPK)_3_U_2_ + AMF	73.0 ± 12.3 ^bc^	7.2 ± 0.8 ^bcd^	11.7 ± 1.3 ^b^	2.7 ± 0.3 ^cd^	1.6 ^c^
OM_600_ + AMF	87.1 ± 22.1 ^abc^	7.8 ± 1.2 ^bc^	22.5 ± 4.1 ^a^	8.4 ± 4.7 ^abc^	1.8^c^
(NPK)_2_U_1_ + OM_200_ +AMF	90.4 ± 17.3 ^abc^	6.6 ± 0.7 ^cd^	21 ± 0.7 ^a^	5.2 ± 2.5 ^abcd^	3.3 ^b^
(NPK)_3_U_2_ + OM_400_ +AMF	77.2 ± 8.1 ^abc^	7.3 ± 0.8 ^bcd^	17.3 ± 3.7 ^ab^	6.1 ± 2.57 ^abcd^	4.2 ^b^
(NPK)_5_U_3_ + OM_600_ +AMF	75.7 ± 31.2 ^abc^	5.3 ± 1.2 ^d^	23.4 ± 5 ^a^	9.9 ± 3.9 ^ab^	1.7 ^c^
Means	79.2 ± 21.9	6.8± 2.1	18.1± 6.31	6.2 ± 3.7	
CV (%)	21.2	17.8	27.8	57.37	
P-value	1.75e-06 <0.001	2.16e-09 <0.001	5.34e-06 <0.001	0.000164 <0.001	

The means that share the same letters within a group in each column are not significantly different by ANOVA and Tukey’s multiple comparison test (p < 0.05).

For stem diameter, analysis of variance and the Tukey test at the 5% threshold identified four distinct groups. The control treatment had the smallest diameter (2.75 mm). The second group included (NPK)_3_U_2_ (5.50 mm) and (NPK)_5_U_4_ + OM_600_ + AMF (5.34 mm). The third group comprised treatments combining NPK, urea, AMF, or organic matter (e.g., (NPK)_3_U_2_ + AMF; OM_600_ + AMF; (NPK)_2_U_1_ + OM_200_ + AMF and (NPK)_3_U_2_ + OM_400_ + AMF), with no significant differences among these treatments. The fourth group, with the largest diameters, included the OM_600_ treatment (9.83 mm) and the AMF treatment (8.88 mm).

Panicle length varied significantly with the fertilization method (**Table 3**). The control treatment recorded the shortest panicle length (5.10 cm), while the longest panicle lengths were observed with AMF treatment (25.2 cm), OM_600_+AMF (22.5 cm), (NPK)2U_1_+OM_200_+AMF (23.4 cm), and (NPK)5U_4_+OM_600_+AMF (21 cm). No significant differences in panicle length were found among these treatments, but significant differences were noted between these treatments and the control.

The colonization of plant roots by indigenous mycorrhizae was influenced by the inoculum (**Table 3**). All treatments, except the control and OM600 treatments, revealed the presence of infectious units. The highest AMF colonization was observed in the AMF treatment (56.43%). In combined treatments, AMF colonization ranged from 1.6% to 4.2%, with the lowest colonization observed in the (NPK)3U_2_ treatment.

### 3.2 Foliar nutrient concentrations

Macro-element concentrations in the shoots of the seedlings after harvest are shown in **Table 4**. The concentrations of N, P, K, Ca, and Mg in pearl millet shoots were influenced by the different treatments. The (NPK)3U_2_ + AMF treatment resulted in the highest N concentration compared to other treatments. Analysis revealed the significant contributions of compost, AMF, and microdosed mineral fertilizers to N, P, K, Ca, and Mg concentrations. The highest foliar P concentration was observed in the OM_600_ treatment, while P concentrations in other treatments did not differ significantly from the control. The most notable differences among fertilized plants were observed in foliar K concentrations. The OM_600_+AMF treatment had significantly higher K concentrations than other treatments. The Ca concentration in inoculated plants decreased significantly compared to other treatments, with the highest Ca concentration found in plants treated with combined microdosed NPK and urea along with AMF. Other treatments did not show significant differences compared to the control. Mg concentrations followed similar trends to Ca, with significant changes only observed in the (NPK)5U_4_ + OM_600_ + AMF treatment.

**Table 4:** Leaf macro-element concentrations of pearl millet seedlings for different treatments.

Treatment	(mg.g^-1^)				
	N	P	K	Ca	Mg
T0	11.5 ± 2.3 ^bc^	5.9 ± 1.8 ^ab^	20.1 ± 4.07 ^c^	4.35 ± 0.2 ^ab ^	4.5 ± 0.6 ^ab^
(NPK)_3_U_2_	10.6 ± 6.0 ^bc^	5.2 ± 0.81 ^ab^	25.3 ± 22.9 ^abc^	6.56 ± 2.3 ^ab ^	4.6 ± 2.6 ^ab^
OM_600_	15.1 ± 5.3 ^c^	7.0 ± 0.7 ^a^	63.9 ± 34.6 ^ab^	4.7 ± 2.2 ^ab ^	4.2 ± 1.9 ^ab^
AMF	5.9 ± 1.0 ^c^	5.7 ± 0.75 ^ab^	17.63 ± 1.3 ^c^	2.9 ± 0.5 ^c ^	2.92 ± 0.5 ^ab^
(NPK)_3_U_2_ + AMF	28.7 ± 0.9 ^a^	3.8 ± 0.4 ^b^	30.78 ± 11.0 ^bc^	8.4 ± 1.02 ^a^	4.2 ± 0.5 ^ab^
OM_600_ + AMF	17.6 ± 4.4 ^b^	4.0 ± 0.6 ^b^	70.0 ± 2.6 ^a^	5.42 ± 1.1 ^ab^	2.3 ± 0.2 ^b^
(NPK)_2_U_1_ + OM_200_ +AMF	13.9 ± 1.6 ^bc^	5.1 ± 0.5 ^ab^	55.4± 3.34 ^abc^	6.0 ± 1.1^ab ^	5.1 ± 0.4^ab^
(NPK)_3_U_2_ + OM_400_ +AMF	18.2 ± 2.8 ^b^	6.40 ± 1.2 ^ab^	44.7 ± 0.8 ^abc^	6.82± 0.8 ^ab ^	4.6 ± 0.9 ^ab^
(NPK)_5_U_4_ + OM_600_ +AMF	10.7 ± 1.3 ^bc^	5.9 ± 0.4 ^ab^	32.3 ± 3.1 ^bc^	6.3 ± 2.3 ^ab ^	5.9 ± 0.4 ^a^
Means	14.7 ± 6.8	5.4 ± 1.2	40.0± 22.1	5.7 ± 1.9	4.2 ± 1.4
CV (%)	23.1	16.9	36.2	26.4	27.6
P-value	1.69e-05 <0.001	0.00841 <0.01	0.00155 <0.01	0.0168 <0.05	0.0463 <0.05

The means that share the same letters within a group in each column are not significantly different by ANOVA and Tukey’s multiple comparison test (p < 0.05).

Micro-element concentrations in the shoots of the seedlings after harvest are presented in **Table 5**. The concentrations of Zn, Cu, Mn, Fe, and Ni in pearl millet shoots varied according to the applied treatment (**Table 5**). The highest foliar Zn concentrations were achieved with combined mineral fertilizers, organic matter, and AMF. Other treatments did not show significant changes compared to the control. Cu concentrations decreased significantly with AMF or (NPK)3U_2_ treatments compared to the control, with no significant differences among other treatments. Mn foliar concentrations were significantly increased by AMF or (NPK)3U_2_ + AMF treatments, while the lowest Mn concentrations were recorded with the OM+AMF treatment. No significant differences were found among other treatments. For Fe concentration, no significant differences were observed between treatments. The highest Ni concentrations were found in OM_600_, AMF, (NPK)2U_1_ + OM200 + AMF, and (NPK)3U_2_ + OM_400_ + AMF treatments, with no significant differences among these treatments or compared to the control.

**Table 5:** Leaf micro-element concentrations of pearl millet seedlings for different treatments.

Treatment	(µg.g^-1^)				
	Zn	Cu	Mn	Fe	Ni
T0	37,3 ± 13.7 ^cd^	6,6 ± 1,4 ^a^	51,8 ± 4,8 ^bc^	309 ± 105,1 ^a^	1,8 ± 0,6 ^b^
(NPK)_3_U_2_	15,1 ± 12 ^d^	2,5 ± 1,3 ^c^	41,6 ± 2,3 ^bc^	297,8 ± 39,64 ^a^	2,7 ± 1,5 ^b^
OM_600_	82,6 ± 44,9 ^c^	6,2 ± 1,2 ^a^	37,3 ± 4,8 ^bc^	420,7 ± 183,34 ^a^	16 ± 6 ^a^
AMF	26,3 ± 3,7 ^d^	2,9 ± 0,2 ^bc^	136,5 ± 16,6 ^a^	276,7 ± 18,71 ^a^	11,6 ± 3,2 ^a^
(NPK)_3_U_2_ + AMF	42,2 ± 4,3 ^cd^	5,7 ± 0,8 ^a^	128,9 ± 42 ^a^	541,2 ± 15,67 ^a^	4,3 ± 0,2 ^b^
OM_600_ + AMF	53,9 ± 8,8^cd^	4,5 ± 0,3 ^abc^	19,4 ± 6,7 ^c^	353,4± 99,65 ^a^	6, ± 1,3 ^b^
(NPK)_2_U_1_ + OM_200_ +AMF	126,8 ± 20,5 ^b^	6,6 ± 0,6 a	38,6 ± 1,9 ^bc^	531 ± 233,22 ^a^	12,5 ± 1,6 ^a^
(NPK)_3_U_2_ + OM_400_ +AMF	166,4 ± 30,9 ^a^	5,4 ± 1,3 a	51,4 ± 4,9 ^bc^	441,8 ± 28,68 ^a^	13,1 ± 1,7 ^a^
(NPK)_5_U_4_ + OM_600_ +AMF	119,9 ± 9,7 b	4,9 ± 0,7 ab	79,1 ± 17,4^ab^	288,2 ± 55,8 ^a^	1,6 ± 0,4 b
Means	74.5 ± 53.3	5 ± 1.6	65± 42.2	384.4 ± 136.90	7.83 ± 5.85
CV (%)	28	19.8	25.4	29.4	32.3
P-value	2.91e-07 <0.001	0.000426 <0.001	2.85e-07 <0.001	0.0498 <0.05	1.62e-06 <0.001

Values that are not followed by the same letters in the same column are significantly different according to Tukey’s test (p < 0.05).

## 4 Discussion

The response of pearl millet to low mineral fertilizer application rates is due to the intrinsic fertility of the soil. Plants could directly acquire sufficient nutrients without their AMF partners after chemical fertilization; while plants might rely on AMF to facilitate the transformation of organic matter following organic fertilization (Liu et al., 2020). This study showed that AMF responded differently to chemical and organic fertilization, suggesting that organic fertilization may activate belowground AMF function to maintain soil nutrients and benefit sustainable agriculture.

The highest root mycorrhizal colonization was achieved with the AMF treatment. The low mycorrhizal fungi colonization at the recommended dose of NPK indicates that the over-application of mineral fertilizers in the pot adversely affects the AMF symbionts, thus reducing the AMF and the benefits provided by the symbionts (Smith and Read, 2010). Many previous studies have shown that the excessive application of chemical fertilizers significantly reduces AMF colonization (Bhadalung et al., 2005; Lin et al., 2012), while other studies have found no significant effect (Chen et al., 2014; Qin et al., 2015) or positive effects (Hazard et al., 2013). In this study, the root colonization rate of the fertilization treatments was significantly lower than that of the no fertilization treatment, ranging from 0.55 to 4.23% (**Table 3**). The root colonization rate was the highest in the AMF treatment and the lowest in the control treatment. It may be that the soil phosphorus level was moderate in the NPK treatment. With the application of the manure treatment or with the combined mineral fertilizer and manure and AMF, there was a possibility that the soil phosphorus levels were too high or too low. Excessive fertilization significantly reduced AMF colonization due to the higher nutrient availability in the soil after the chemical addition (Hack et al., 2019; Zhang et al., 2021). According to Liu et al. (2020) and Westphal et al. (2008), after the application of the chemical fertilizers, the plant can directly absorb enough nutrients to reduce its dependence on AMF symbiosis. The plant reduces the allocation of photosynthates and reduces the AMF colonization after the application of chemical fertilizers (Trejo et al., 2021).

The performance of AMF combined with different doses of microdosed fertilizers led to significant changes in plant growth parameters. The positive effect of mineral fertilizer was obtained with (NPK)_3_U_2_ treatment compared to the control. Plant development and growth conditions were improved by organo-mineral fertilization. This stimulation of growth and development could be linked to a more satisfactory mineral nutrition of the millet with the addition of fertilizer. The positive effect of organo-mineral fertilization on millet development and yield has been reported in the literature (Ndiaye et al., 2019; Pale et al., 2021). Moreover, this effect is more pronounced with the combination of microdosed fertilizer and AMF [(NPK)_3_U_2_ + AMF]. Reducing the use of microdosed fertilizer combined with AMF improved growth parameters. This result is significant considering that the improvement in growth parameters was achieved by significantly reducing fertilization. It also helps to demonstrate the environmental benefits of using AMF to improve plant millet growth. The highest growth parameters were obtained with AMF or OM_600_ treatments. These observations confirm that OM is also a suitable product for soil fertilization. Some previous studies reported an additive effect when combining FM with OM (Bielders and Gérard, 2015; Ibrahim et al., 2015; Somda et al., 2017; Tovihoudji et al., 2019). In contrast, other previous studies have shown that negative interactions between organic amendments and fertilizers are most often observed (Chivenge et al., 2011; Ouedraogo et al., 2021). Indeed, several studies have shown that nutrients from organic matter sources such as crop residues, compost and manure can stimulate mycorrhization (Panja and Chaudhuri, 2004; Gosling et al., 2006; Jan et al., 2014). However, a comparative study of the results obtained with the addition of organic matter combined with AMF (OM_600_ + AMF) or without AMF showed that the AMF had no effect on millet growth. These results can be explained by the fact that the soil fertility provided by the manure reduced the mycorrhizal potential by affecting AMF development. These results are corroborated by those of Gosling et al. (2006), who states that the effect of organic amendments on mycorrhization is unpredictable because amendments with a high phosphorus concentration can lead to a decrease in mycorrhizal abundance.

The impact of the microdosing fertilizer applied to millet was tested with fertilizer doses respectively lower, equal, or higher than recommended, combined with AMF and manure. The results show that higher fertilizer doses reduce growth parameters. Furthermore, the combination of organic and mineral fertilization in microdosing, which is not favorable for AMF, likely explains the low root infection levels. This suggests that microdosing with both types of fertilizers may not benefit AMF. These results corroborate those of Ricardos et al. (2020), who obtained a greenhouse mycorrhization frequency of less than 45% in maize (*Zea mays* L.). They explained this low mycorrhization by the low oxygen concentration around the roots confined in the pots.

Nutrients are the determining factors for pearl millet growth and productivity under Sahelian conditions. In this study, AMF and amendment treatments were the most efficient fertilizers in terms of growth parameters under greenhouse conditions. Compost amendment significantly increased the growth parameters. Other researchers have shown a significant effect of compost and AMF complex on plant growth in a greenhouse experiment, where the root colonization and root dry weight were improved (Akhter et al., 2015). AMF and compost have been used to improve the plant growth and mineral nutrition of many species (Mrabet et al., 2014), where a full and different dose of compost inoculated with AMF significantly increased root and shoot biomass (Jan et al., 2014). The use of compost and AMF has also increased growth parameters (Shah et al., 2022). In our study, we assessed for the first time the effect of AMF and microdosing of fertilizer and compost under greenhouse conditions. Small-seeded cereals such as pearl millet require early nutrient supply as the plants will exhaust the seed reserves of nutrients 8–10 days after sowing and all flower parts are formed 30 days after sowing (Rebafka et al., 1993). However, it is well established that nitrogen and phosphorus are the most limiting nutrients in most agricultural soils in sub-Saharan Africa (Kurwakumire et al., 2014; Stewart et al., 2020). Our results demonstrated the efficiency of this combination in increasing mineral nutrition. Leaf mineral nutrition, especially macroelements, efficiently enhanced the effectiveness of microdosing with combined AMF and compost on plant growth. Muchow and Davis (1988) reported that high NPK rates stimulated pearl millet leaf growth, resulting in higher nutrient uptake. Nitrogen and phosphorus uptake was consistently higher. As a result, pearl millet dry matter production and hence nutrient uptake were significantly reduced. The NPK+AMF recommended rate was likely to have taken up more nitrogen and phosphorus than the NPK microdose due to higher nitrogen and phosphorus concentrations. Microdosing of fertilizer increased the branching of pearl millet fibrous root systems, which increased water uptake. This also improved nutrient availability which increased nutrient uptake by pearl millet and thus increased growth and biomass accumulation. Ibrahim et al. (2015) observed that pearl millet crops that received nitrogen and phosphorus at optimal rates were able to grow at their maximum potential, thus increasing N and P uptake. The same conclusion was also reached by Bationo et al. (2005). In contrast, AMF increased the uptake of microelements such as Mn and Ni compared to the control plants. The mixture of microdosed fertilizer and AMF contributed to the uptake of another microelement, Mn. However, the low root colonization in the substrate supplemented with compost compared to the AMF strain alone could be explained by the high availability of macro and micro-nutrients added to the soil via the compost. In particular, the phosphorus may have reduced root colonization. This result confirmed the negative correlation found between the amount of phosphorus and the colonization rate of millet (Bouamri et al., 2006). In addition, the colonization level depended on the composition and maturity of the compost, such as that found in a *Zea mays* culture (Cozzolino et al., 2016), where compost of 60 and 120 days of maturity negatively affected plant growth and root colonization in greenhouse conditions. Macro and micronutrient concentrations could partly depend not only on the fungal partner but also on the host plant (Ravnskov and Jakobsen, 1995). The most common and well-known interaction of P with micronutrients is the interaction of P with Zn, which can lead to reduction of the concentration of Zn in plants under conditions of excessive P (Barben et al., 2010). AMF and compost have been used to improve the plant growth and mineral nutrition of many species (Mrabet et al., 2014). The combination of compost and mineral fertilizer and AMF seems to favor Zn and Ni over Cu, Fe, and Mn.

## 5 Conclusion

Microdosing reduced the amount of mineral fertilizer used, improved growth parameters, and increased fertilizer efficiency. However, this practice seems to reduce the mycorrhizal potential by affecting the development of vesicular and arbuscular mycorrhizal fungi. The results of the present study showed that millet production could be further improved by combining inorganic fertilizers with manure application. The highest growth parameters were obtained with combined application of organic matter and NPK. N and Ca concentrations can be improved by using AMF and microdosing NPK, while their combination with organic matter mainly improved K concentration.

## Data Availability

The original contributions presented in the study are included in the article/supplementary material, further inquiries can be directed to the corresponding author/s.

## References

[B1] Adesemoye A. O. Kloepper J. W. (2009). Plant–microbes interactions in enhanced fertilizer-use efficiency. Appl. Microbiol. Biotechnol. 85, 1–12. doi: 10.1007/s00253-009-2196-0 19707753

[B2] Adesemoye A. O. Torbert H. A. Kloepper J. W. (2008). Enhanced plant nutrient use efficiency with PGPR and AMF in an integrated nutrient management system. Can. J. Microbiol. 54, 876–886. doi: 10.1139/W08-081 18923557

[B3] Adoko M. Y. Noumavo A. D. P. Agbodjato N. A. Amogou O. Salami H. A. Aguégué R. M.. (2022). Effect of the application or coating of PGPR-based biostimulant on the growth, yield and nutritional status of maize in benin. Front. Plant Sci. 13, 1064710. 36578347 10.3389/fpls.2022.1064710 PMC9791037

[B4] Affholder F. Poeydebat C. Corbeels M. Scopel E. Tittonell P. (2013). The yield gap of major food crops in family agriculture in the tropics: Assessment and analysis through field surveys and modelling. Field Crops Res. 143, 106–118.

[B5] Akhter A. Hage-Ahmed K. Soja G. Steinkellner S. (2015). Compost and biochar alter mycorrhization, tomato root exudation, and development of *Fusarium oxysporum* f. sp. lycopersici. Front. Plant Sci. 6. doi: 10.3389/fpls.2015.00529 PMC4498038 26217373

[B6] Alori E. T. Babalola O. O. (2018). Microbial inoculants for improving crop quality and human health in africa. Front. Microbiol. 9, 2213. 30283427 10.3389/fmicb.2018.02213 PMC6156547

[B7] Aune J. B. Bationo A. (2008). Agricultural intensification in the Sahel – The ladder approach. Agric. Syst. 98, 119–125. doi: 10.1016/j.agsy.2008.05.002

[B8] Barben S. A. Hopkins B. G. Jolley V. D. Webb B. L. Nichols B. A. (2010). Phosphorus and zinc interactions in chelator-buffered solution grown russet burbank potato. J. Plant Nutr. 33, 587–601. doi: 10.1080/01904160903506308

[B9] Bastos L. M. Faye A. Stewart Z. P. Akplo T. M. Min D. Prasad P. V. V.. (2022). Variety and management selection to optimize pearl millet yield and profit in Senegal. Eur. J. Agron. 139, 126565. doi: 10.1016/j.eja.2022.126565

[B10] Bationo A. Kihara J. Waswa B. Ouattara B. Vanlauwe B. (2005). Technologies for sustainable management of sandy sahelian soils. Manage. Trop. Sandy Soils Sustain. Agric., 414.

[B11] Bhadalung N. N. Suwanarit A. Dell B. Nopamornbodi O. Thamchaipenet A. Rungchuang J. (2005). Effects of long-term NP-fertilization on abundance and diversity of arbuscular mycorrhizal fungi under a maize cropping system. Plant Soil 270, 371–382. doi: 10.1007/s11104-004-1829-4

[B12] Bielders C. L. Gérard B. (2015). Millet response to microdose fertilization in south–western Niger: Effect of antecedent fertility management and environmental factors. Field Crops Res. 171, 165–175. doi: 10.1016/j.fcr.2014.10.008

[B13] Blessing O. C. Ibrahim A. Safo E. Y. Yeboah E. Robert C. Logah V.. (2017). Fertilizer micro-dosing in West African low-input cereals cropping: Benefits, challenges and improvement strategies. Afr. J. Agric. Res. 12, 1169–1176. doi: 10.5897/ajar2016.11559

[B14] Bouamri R. Dalpé Y. Serrhini M. N. Bennani A. (2006). Arbuscular mycorrhizal fungi species associated with rhizosphere of phoenix dactylifera l. @ in morocco. Afr. J. Biotechnol. 5 (6), 510–516.

[B15] Bucher M. (2007). Functional biology of plant phosphate uptake at root and mycorrhiza interfaces. New Phytol. 173, 11–26. doi: 10.1111/j.1469-8137.2006.01935.x 17176390

[B16] Chaudhary S. Dheri G. S. Brar B. S. (2017). Long-term effects of NPK fertilizers and organic manures on carbon stabilization and management index under rice-wheat cropping system. Soil Tillage Res. 166, 59–66.

[B17] Chen Y.-L. Zhang X. Ye J.-S. Han H.-Y. Wan S.-Q. Chen B. D. (2014). Six-year fertilization modifies the biodiversity of arbuscular mycorrhizal fungi in a temperate steppe in Inner Mongolia. Soil Biol. Biochem. 69, 371–381. doi: 10.1016/j.soilbio.2013.11.020

[B18] Chivenge P. Vanlauwe B. Six J. (2011). Does the combined application of organic and mineral nutrient sources influence maize productivity? A meta-analysis. Plant Soil 342, 1–30. doi: 10.1007/s11104-010-0626-5

[B19] Compant S. Samad A. Faist H. Sessitsch A. (2019). A review on the plant microbiome: Ecology, functions, and emerging trends in microbial application. J. Adv. Res. 19, 29–37. doi: 10.1016/j.jare.2019.03.004 31341667 PMC6630030

[B20] Cozzolino V. Di Meo V. Monda H. Spaccini R. Piccolo A. (2016). The molecular characteristics of compost affect plant growth, arbuscular mycorrhizal fungi, and soil microbial community composition. Biol. Fertil Soils 52, 15–29. doi: 10.1007/s00374-015-1046-8

[B21] Debieu M. Sine B. Passot S. Grondin A. Akata E. Gangashetty P.. (2018). Response to early drought stress and identification of QTLs controlling biomass production under drought in pearl millet. PloS One 13, e0201635. doi: 10.1371/journal.pone.0201635 30359386 PMC6201870

[B22] Defrance D. Sultan B. Castets M. Famien A. M. Baron C. (2020). Impact of climate change in west africa on cereal production per capita in 2050. Sustainability 12 (18), 7585.

[B23] Diatta A. A. Abaye O. Thomason W. E. Lo M. Thompson T. L. Vaughan L. J.. (2020). Evaluating pearl millet and mungbean intercropping in the semi-arid regions of senegal. Agron. J. 112 (5), 4451–4466.

[B24] Djanaguiraman M. Perumal R. Ciampitti I. A. Gupta S. K. Prasad P. V. V. (2018). Quantifying pearl millet response to high temperature stress: thresholds, sensitive stages, genetic variability and relative sensitivity of pollen and pistil. Plant Cell Environ. 41 (5), 993–1007. 28173611 10.1111/pce.12931

[B25] Faiz M. A. Bana R. S. Choudhary A. K. Laing A. M. Bansal R. Bhatia A.. (2022). Zero tillage, residue retention and system-intensification with legumes for enhanced pearl millet productivity and mineral biofortification. Sustainability 14 (1), 543.

[B26] Fall A. F. Nakabonge G. Ssekandi J. Founoune-Mboup H. Badji A. Ndiaye A.. (2023). Combined effects of indigenous arbuscular mycorrhizal fungi (AMF) and NPK fertilizer on growth and yields of maize and soil nutrient availability. Sustainability 15, 2243. doi: 10.3390/su15032243

[B27] Fasusi O. A. Cruz C. Babalola O. O. (2021). Agricultural sustainability: microbial biofertilizers in rhizosphere management. Agriculture 11, 163. doi: 10.3390/agriculture11020163

[B28] Faye A. Akplo T. M. Stewart Z. P. Min D. Obour A. K. Assefa Y.. (2023). Increasing millet planting density with appropriate fertilizer to enhance productivity and system resilience in senegal. Sustainability 15 (5), 4093.

[B29] Ganyo K. K. Muller B. Ndiaye M. Gaglo E. K. Guissé A. Adam M. (2019). Defining fertilization strategies for sorghum (Sorghum bicolor (L.) moench) production under sudano-sahelian conditions: Options for late basal fertilizer application. Agronomy 9 (11), 697.

[B30] Genre A. Lanfranco L. Perotto S. Bonfante P. (2020). Unique and common traits in mycorrhizal symbioses. Nat. Rev. Microbiol. 18 (11), 649–660. 32694620 10.1038/s41579-020-0402-3

[B31] Giovannetti M. Mosse B. (1980). An evaluation of techniques for measuring vesicular arbuscular mycorrhizal infection in roots. New Phytol., 489–500.

[B32] Ghobadi M. Movahhedi Dehnavi M. Yadavi A. R. Parvizi K. Zafari D. (2020). Reduced P fertilization improves Fe and Zn uptake in potato when inoculated with AMF in P, Fe and Zn deficient soil. Rhizosphere 15, 100239. doi: 10.1016/j.rhisph.2020.100239

[B33] Gosling P. Hodge A. Goodlass G. Bending G. D. (2006). Arbuscular mycorrhizal fungi and organic farming. Agric. Ecosyst. Environ. 113, 17–35. doi: 10.1016/j.agee.2005.09.009

[B34] Hack C. M. Porta M. Schäufele R. Grimoldi A. A. (2019). Arbuscular mycorrhiza mediated effects on growth, mineral nutrition and biological nitrogen fixation of *Melilotus alba* Med. in a subtropical grassland soil. Appl. Soil Ecol. 134, 38–44. doi: 10.1016/j.apsoil.2018.10.008

[B35] Haussmann B. I. G. Boureima S. S. Kassari I. A. Moumouni K. H. Boubacar A. (2007). Mechanisms of adaptation to climate variability in west african pearl millet landraces–a preliminary. J. SAT Agric. Res. 3 (1), 1–3.

[B36] Hazard C. Gosling P. van der Gast C. J. Mitchell D. T. Doohan F. M. Bending G. D. (2013). The role of local environment and geographical distance in determining community composition of arbuscular mycorrhizal fungi at the landscape scale. ISME J. 7, 498–508. doi: 10.1038/ismej.2012.127 23096401 PMC3578569

[B37] Ibrahim A. Abaidoo R. C. Fatondji D. Opoku A. (2015). Hill placement of manure and fertilizer micro-dosing improves yield and water use efficiency in the Sahelian low input millet-based cropping system. Field Crops Res. 180, 29–36. doi: 10.1016/j.fcr.2015.04.022

[B38] Jan B. Ali A. Wahid F. Shah S. N. M. Khan A. Khan F. (2014). Effect of arbuscular mycorrhiza fungal inoculation with compost on yield and phosphorous uptake of berseem in alkaline calcareous soil. Am. J. Plant Sci. 05, 1359–1369. doi: 10.4236/ajps.2014.59150

[B39] Kurwakumire N. Chikowo R. Mtambanengwe F. Mapfumo P. Snapp S. Johnston A.. (2014). Maize productivity and nutrient and water use efficiencies across soil fertility domains on smallholder farms in Zimbabwe. Field Crops Res. 164, 136–147. doi: 10.1016/j.fcr.2014.05.013

[B40] Lin X. Feng Y. Zhang H. Chen R. Wang J. Zhang J.. (2012). Long-term balanced fertilization decreases arbuscular mycorrhizal fungal diversity in an arable soil in north China revealed by 454 pyrosequencing. Environ. Sci. Technol. 46, 5764–5771. doi: 10.1021/es3001695 22582875

[B41] Liu J. Zhang J. Li D. Xu C. Xiang X. (2020). Differential responses of arbuscular mycorrhizal fungal communities to mineral and organic fertilization. MicrobiologyOpen 9, e00920. doi: 10.1002/mbo3.920 31397116 PMC6957387

[B42] Manssour A. M. Abdoulkadri L. Haboubacar M. B. A. Djibo E. S. Ali A. Zoubeirou A. M. Impact de la Combinaison Regeneration Naturelle Assistee (Rna) Et Engrais En Microdose Sur La Productivite Du Mil (Pennisetum Glaucum (L.) R. Br.) Au Niger.

[B43] Meena R. Kumar S. Datta R. Lal R. Vijayakumar V. Brtnicky M.. (2020). Impact of agrochemicals on soil microbiota and management. A Review Land. 9, 34. doi: 10.3390/land9020034

[B44] Monerie P. A. Sanchez-Gomez E. Gaetani M. Mohino E. Dong B. (2020). Future evolution of the sahel precipitation zonal contrast in CESM1. Climate Dynamics 55 (9), 2801–2821.

[B45] Mrabet S. E. Ouahmane L. Mousadik A. E. Msanda F. Abbas Y. (2014). The effectiveness of arbuscular mycorrhizal inoculation and bio-compost addition for enhancing reforestation with *Argania spinosa* in Morocco. Open J. For. 04, 14–23. doi: 10.4236/ojf.2014.41003

[B46] Muchow R. C. Davis R. (1988). Effect of nitrogen supply on the comparative productivity of maize and sorghum in a semi-arid tropical environment II. Radiation interception and biomass accumulation. Field Crops Res. 18, 17–30. doi: 10.1016/0378-4290(88)90056-1

[B47] Mulyadi Jiang L. (2023). Combined application of arbuscular mycorrhizal fungi (AMF) and nitrogen fertilizer alters the physicochemical soil properties, nitrogen uptake, and rice yield in a polybag experiment. Agriculture 13, 1364. doi: 10.3390/agriculture13071364

[B48] Ndiaye A. Ndiaye O. Bamba B. Guèye M. Sawané O. (2019). Effets de la fertilisation organo- minérale sur la croissance et le rendement du « mil sanio » (*Pennisetum glaucum* L. R. Br) en Haute Casamance (Sénégal). Eur. Sci. J. ESJ. 15 (33), 155. doi: 10.19044/esj.2019.v15n33p155

[B49] Ndour P. M. S. Barry C. M. Tine D. de la Fuente Cantó C. Gueye M. Barakat M.. (2021). Pearl millet genotype impacts microbial diversity and enzymatic activities in relation to root-adhering soil aggregation. Plant Soil 464, 109–129. doi: 10.1007/s11104-021-04917-w

[B50] Ouedraogo J. Serme I. Pouya M. B. Sanon S. B. Ouattara K. Lompo F. (2021). Improvement of sorghum productivity through introducing integrated soil fertility management options in the Northern Sudanian zone of Burkina Faso. Int. J. Biol. Chem. Sci. 14, 3262–3274. doi: 10.4314/ijbcs.v14i9.23

[B51] Pale S. Barro A. Koumbem M. Sere A. Traore H. (2021). Effets du travail du sol et de la fertilisation organo-minérale sur les rendements du mil en zone soudano-sahélienne du Burkina Faso. Int. J. Biol. Chem. Sci. 15, 497–510. doi: 10.4314/ijbcs.v15i2.10

[B52] Pan X. Lv J. Dyck M. He H. (2021). Bibliometric analysis of soil nutrient research between 1992 and 2020. Agriculture 11 (3), 223.

[B53] Panja B. N. Chaudhuri S. (2004). Exploitation of soil arbuscular mycorrhizal potential for AM-dependent mandarin orange plants by pre-cropping with mycotrophic crops. Appl. Soil Ecol. 26, 249–255. doi: 10.1016/j.apsoil.2003.12.007

[B54] Passot S. Gnacko F. Moukouanga D. Lucas M. Guyomarc’h S. Ortega B. M.. (2016). Characterization of pearl millet root architecture and anatomy reveals three types of lateral roots. Front. Plant Sci. 7, 829. 27379124 10.3389/fpls.2016.00829 PMC4904005

[B55] Pellegrino E. Öpik M. Bonari E. Ercoli L. (2015). Responses of wheat to arbuscular mycorrhizal fungi: a meta-analysis of field studies from 1975 to 2013. Soil Biol. Biochem. 84, 210–217.

[B56] Phillips J. M. Hayman D. S. (1970). Improved procedures for clearing roots and staining parasitic and vesicular-arbuscular mycorrhizal fungi for rapid assessment of infection. Trans. Br. mycological Soc. 55 (1), 158–IN18.

[B57] Prasad P. V. Bheemanahalli R. Jagadish S. K. (2017). Field crops and the fear of heat stress–opportunities, challenges and future directions. Field Crops Res. 200, 114–121.

[B58] Pucher A. Høgh-Jensen H. Gondah J. Hash C. T. Haussmann B. I. G. (2014). Micronutrient density and stability in west African pearl millet—Potential for biofortification. Crop Sci. 54, 1709–1720. doi: 10.2135/cropsci2013.11.0744

[B59] Qin H. Lu K. Strong P. J. Xu Q. Wu Q. Xu Z.. (2015). Long-term fertilizer application effects on the soil, root arbuscular mycorrhizal fungi and community composition in rotation agriculture. Appl. Soil Ecol. 89, 35–43. doi: 10.1016/j.apsoil.2015.01.008

[B60] Ravnskov S. Jakobsen I. (1995). Functional compatibility in arbuscular mycorrhizas measured as hyphal P transport to the plant. New Phytol. 129, 611–618. doi: 10.1111/j.1469-8137.1995.tb03029.x

[B61] Rebafka F.-P. Bationo A. Marschner H. (1993). Phosphorus seed coating increases phosphorus uptake, early growth and yield of pearl millet (*Pennisetum glaucum* (L.) R. Br.) grown on an acid sandy soil in Niger, West Africa. Fertil Res. 35, 151–160. doi: 10.1007/BF00750633

[B62] Ricardos A. Nestor A. Nadège A. Pacôme N. Marcellin A. Nicodeme C. (2020). Greenhouse evaluation of the growth of *Zea mays* L. inoculated by arbuscular mycorrhizal fungi strains in native arbuscules on ferrous soil. J. Agric. Crop Res. 8, 55–63. doi: 10.33495/jacr_v8i3.20.106

[B63] Saba F. Taonda S. J. B. Serme I. Bandaogo A. A. Sourwema A. P. Kabre A. (2017). Effets de la microdose sur la production du niébé, du mil et du sorgho en fonction la toposéquence. Int. J. Biol. Chem. Sci. 11 (5), 2082–2092.

[B64] Schaefer D. A. Gui H. Mortimer P. E. Xu J. (2021). Arbuscular mycorrhiza and sustainable agriculture. Circular Agric. Syst. 1 (1), 1–7.

[B65] Schütz L. Gattinger A. Meier M. Müller A. Boller T. Mäder P.. (2018). Improving crop yield and nutrient use efficiency *via* biofertilization–a global meta-analysis. Front. Plant Sci. 8, 2204. 29375594 10.3389/fpls.2017.02204 PMC5770357

[B66] Selim M. M. (2020). Introduction to the integrated nutrient management strategies and their contribution to yield and soil properties. Int. J. Agron. 2020, 1–14. doi: 10.1155/2020/2821678

[B67] Serba D. D. Yadav R. S. (2016). Genomic tools in pearl millet breeding for drought tolerance: status and prospects. Front. Plant Sci. 7, 1724. 27920783 10.3389/fpls.2016.01724 PMC5118443

[B68] Shah A. H. Laishram N. Singh A. (2022). Effect of mycorhizal and vermicompost application on growth and quality flower production of annual chrysanthemum (*Chrysanthemum coronium* L.). Int. J. Plant Soil Sci. 34 (17), 114–123. doi: 10.9734/ijpss/2022/v34i1731043

[B69] Shanmugavel D. Rusyn I. Solorza-Feria O. Sathish-Kumar K. (2023). Sustainable SMART fertilizers in agriculture systems: A review on fundamentals to in-field applications. Sci. Total Environ., 166729. 37678530 10.1016/j.scitotenv.2023.166729

[B70] Simpson R. J. Oberson A. Culvenor R. A. Ryan M. H. Veneklaas E. J. Lambers H.. (2011). Strategies and agronomic interventions to improve the phosphorus-use efficiency of farming systems. Plant Soil 349, 89–120. doi: 10.1007/s11104-011-0880-1

[B71] Smith F. A. Smith S. E. (2011). What is the significance of the arbuscular mycorrhizal colonisation of many economically important crop plants? Plant Soil 348, 63–79. doi: 10.1007/s11104-011-0865-0

[B72] Smith S. E. Read D. J. (2010). Mycorrhizal symbiosis (Academic Press).

[B73] Somda B. B. Ouattara B. Serme I. Pouya M. B. Lompo F. Taonda S. J. B.. (2017). Détermination des doses optimales de fumures organo-minérales en microdose dans la zone soudano-sahélienne du Burkina Faso. Int. J. Biol. Chem. Sci. 11, 670. doi: 10.4314/ijbcs.v11i2.11

[B74] Stewart Z. P. Pierzynski G. M. Middendorf B. J. Prasad P. V. V. (2020). Approaches to improve soil fertility in sub-Saharan Africa. J. Exp. Bot. 71, 632–641. doi: 10.1093/jxb/erz446 31586430 PMC6946000

[B75] Suman J. Rakshit A. Ogireddy S. D. Singh S. Gupta C. Chandrakala J. (2022). Microbiome as a key player in sustainable agriculture and human health. Front. Soil Sci. 2, 821589.

[B76] Supriya A. Senthilvel S. Nepolean T. Eshwar K. Rajaram V. Shaw R.. (2011). Development of a molecular linkage map of pearl millet integrating DArT and SSR markers. Theor. Appl. Genet. 123, 239–250. 21476042 10.1007/s00122-011-1580-1

[B77] Syed Ab Rahman S. F. Singh E. Pieterse C. M. J. Schenk P. M. (2018). Emerging microbial biocontrol strategies for plant pathogens. Plant Sci. 267, 102–111. doi: 10.1016/j.plantsci.2017.11.012 29362088

[B78] Thirkell T. J. Charters M. D. Elliott A. J. Sait S. M. Field K. J. (2017). Are mycorrhizal fungi our sustainable saviours? considerations for achieving food security. J. Ecol. 105 (4), 921–929.

[B79] Tounkara A. Clermont-Dauphin C. Affholder F. Ndiaye S. Masse D. Cournac L. (2020). Inorganic fertilizer use efficiency of millet crop increased with organic fertilizer application in rainfed agriculture on smallholdings in central Senegal. Agric. Ecosyst. Environ. 294, 106878. doi: 10.1016/j.agee.2020.106878

[B80] Tovihoudji P. G. Akponikpè P. B. I. Agbossou E. K. Bielders C. L. (2019). Variability in maize yield and profitability following hill-placement of reduced mineral fertilizer and manure rates under smallholder farm conditions in northern Benin. Field Crops Res. 230, 139–150. doi: 10.1016/j.fcr.2018.10.018

[B81] Trejo D. Sangabriel-Conde W. Gavito-Pardo M. E. Banuelos J. (2021). Mycorrhizal inoculation and chemical fertilizer interactions in pineapple under field conditions. Agriculture 11, 934. doi: 10.3390/agriculture11100934

[B82] Tshibangu Kazadi A. Baert G. Lwalaba Wa Lwalaba J. Kirika Ansey B. Haesaert G. Mukobo Mundende R.-P. (2020). Increasing of NPK fertilizer efficiency by arbuscular mycorrhiza in common bean (*Phaseolus vulgaris* L.). Gesunde Pflanz 72, 303–310. doi: 10.1007/s10343-020-00513-7

[B83] Twomlow S. Rohrbach D. Dimes J. Rusike J. Mupangwa W. Ncube B.. (2010). Micro-dosing as a pathway to Africa’s Green Revolution: evidence from broad-scale on-farm trials. Nutr. Cycl Agroecosyst 88, 3–15. doi: 10.1007/s10705-008-9200-4

[B84] Ullah A. Ahmad A. Khaliq T. Akhtar J. (2017). Recognizing production options for pearl millet in pakistan under changing climate scenarios. J. Integr. Agric. 16 (4), 762–773.

[B85] Vadez V. Hash T. Bidinger F. R. Kholova J. (2012). II.1.5 Phenotyping pearl millet for adaptation to drought. Front. Physiol. 3. doi: 10.3389/fphys.2012.00386 PMC3475983 23091462

[B86] Wang W. Shi J. Xie Q. Jiang Y. Yu N. Wang E. (2017). Nutrient exchange and regulation in arbuscular mycorrhizal symbiosis. Mol. Plant 10 (9), 1147–1158. 28782719 10.1016/j.molp.2017.07.012

[B87] Westphal A. Snyder N. L. Xing L. Camberato J. J. (2008). Effects of inoculations with mycorrhizal fungi of soil less potting mixes during transplant production on watermelon growth and early fruit yield. HortScience 43, 354–360. doi: 10.21273/HORTSCI.43.2.354

[B88] Zhang S. Luo P. Yang J. Irfan M. Dai J. An N.. (2021). Responses of arbuscular mycorrhizal fungi diversity and community to 41-year rotation fertilization in brown soil region of northeast China. Front. Microbiol 12. doi: 10.3389/fmicb.2021.742651 PMC8542923 34707593

[B89] Zhang S. Lehmann A. Zheng W. You Z. Rillig M. C. (2019). Arbuscular mycorrhizal fungi increase grain yields: a meta-analysis. New Phytol. 222 (1), 543–555. 30372522 10.1111/nph.15570

[B90] Ziane H. Hamza N. Meddad-Hamza A. (2021). Arbuscular mycorrhizal fungi and fertilization rates optimize tomato (*Solanum lycopersicum* L.) growth and yield in a Mediterranean agroecosystem. J. Saudi Soc Agric. Sci. 20, 454–458. doi: 10.1016/j.jssas.2021.05.009

